# Experimental support for partial compensation, not matched, partner response rules in blue tits

**DOI:** 10.1093/beheco/arae059

**Published:** 2024-12-07

**Authors:** Aisha C Bründl, Léa A Lejeune, Purabi Deshpande, Jessica E Mulvey, Alice C Thiney, Alexis S Chaine, Andrew F Russell

**Affiliations:** Station d’Ecologie Théorique et Expérimentale du CNRS (UAR2029), 2 route du CNRS, 09200, Moulis, France; Centre for Ecology and Conservation, University of Exeter, Treliever Road, Penryn, Cornwall TR10 9FE, United Kingdom; Station d’Ecologie Théorique et Expérimentale du CNRS (UAR2029), 2 route du CNRS, 09200, Moulis, France; Centre for Ecology and Conservation, University of Exeter, Treliever Road, Penryn, Cornwall TR10 9FE, United Kingdom; Centre for Ecology and Conservation, University of Exeter, Treliever Road, Penryn, Cornwall TR10 9FE, United Kingdom; Station d’Ecologie Théorique et Expérimentale du CNRS (UAR2029), 2 route du CNRS, 09200, Moulis, France; Station d’Ecologie Théorique et Expérimentale du CNRS (UAR2029), 2 route du CNRS, 09200, Moulis, France; Centre for Ecology and Conservation, University of Exeter, Treliever Road, Penryn, Cornwall TR10 9FE, United Kingdom

**Keywords:** bi-parental care, incomplete compensation, parental investment, provisioning rules, sexual conflict

## Abstract

Outcomes of theoretical models on conflict resolution between investing partners in bi-parental care systems typically predict "partial compensation" or "matched" response rules, depending on underlying assumptions. Further, although experimental tests in birds suggest that care levels by pair members are largely associated with partial compensation responses, this outcome partly depends on the type of experiment used and its effects on model assumptions. To elucidate support for both the underlying assumptions and predictions of models predicting partner compensation versus matching, we performed temporary, bi-directional brood size manipulations during late nestling provisioning in blue tits (*Cyanistes caeruleus*) in the French Pyrenees. We found support for assumptions of both compensation and matching models. For example, females spent more time with the brood than males, leading to potential information asymmetries on brood demand as expected by matching models. Nevertheless, both pair members changed their provisioning comparably in response to brood size manipulations, suggesting that pair members have comparable cost-benefit functions in response to variation in brood demand, as assumed under partial compensation models. Despite support for the underlying assumptions of both models, we primarily found support for predictions of partial compensation models in provisioning responses. Notably, modest changes by one member of the pair on experimental days were met with larger changes by the other, after controlling for brood size and provisioning rates on control days. Our results corroborate previous findings in blue tits using alternative experimental approaches. We suggest that future studies could benefit from understanding when partial compensation responses dominate matched ones, despite apparent asymmetries in information over brood demand.

## Teaser

When both parents care for their offspring, conflict is expected over their relative contributions. To investigate how such conflict might be resolved, we changed the number of nestlings in broods of blue tits. Although both parents responded, low-responding parents had higher-responding partners, consistent with models suggesting that conflict is resolved by partial compensation response rules. Further work is required to discover whether parents respond to each other’s contributions directly or indirectly to changes in the number and begging intensity of offspring.

## Introduction

Bi-parental care is taxonomically widespread and is the predominant care system in birds (Royle et al. 2012). However, because contributing to offspring care is costly, each pair member would benefit from their unrelated partner contributing more than their “fair” share (Trivers 1972). Consequently, for bi-parental care to evolve and be maintained by selection, sexual conflict over relative contributions to care needs to be sufficiently resolved. Depending on the underlying assumptions, theoretical models demonstrate various solutions to this conflict. For example, when one member of a pair reduces investment in care, the other can do the same (matched response) or it can increase investment, either by an equivalent amount (full compensation) or by a proportion (partial compensation) (Houston and Davies 1985; McNamara et al. 1999; Smith and Härdling 2000; Johnstone and Hinde 2006; Savage et al. 2013). A meta-analysis of partner response rules in manipulative studies of birds showed significant, but not universal, support for partial compensation (Harrison et al. 2009). Further, experimental design was a significant contributor to variation in responses, clouding our understanding of the relative support for competing models of bi-parental care evolution. Clarifying support for competing models of bi-parental care thus requires simultaneous tests of multiple model assumptions and predictions within manipulative studies.

Variable response rules in theoretical models largely stem from contrasting assumptions. For example, models showing partial compensation outcomes assume that both members of the pair have comparable cost-benefit functions in investing in the specific form of care measured (Houston and Davies 1985; McNamara et al. 1999) and comparable information about brood demand (Johnstone and Hinde 2006). However, neither need be the case. First, under high paternity uncertainty or where males contribute to other activities, such as territorial defense, males might be selected to have fixed investment in brood care and to be non-responsive to changes by females (Dearborn 2001; Kilner 2002; Trnka and Grim 2013). Alternatively, if high pre-natal investment by females increases the slope of the cost function of post-natal care (Heaney and Monaghan 1995; Monaghan et al. 1998; Visser and Lessells 2001) and concomitantly increases the benefits function of within-pair investment for males (Grundel 1987; Beissinger 1990; Wright and Cuthill 1990), then full compensation responses are possible (Smith and Härdling 2000; Savage et al. 2013). Second, even if assumptions regarding comparable cost-benefit functions between pair members are met, stable solutions to bi-parental care can be influenced by asymmetries in information over offspring value or demand. For example, when one member of the pair has more information, then it can pay the less informed partner to match (rather than compensate) the investment patterns of the more informed partner (Johnstone and Hinde 2006). Thus, a potential reason for significant associations between the type of manipulation performed and response rules observed might stem from inadvertent impacts of manipulation form on model assumptions (Harrison et al. 2009).

Despite this possibility, studies rarely test whether manipulations influence model assumptions, except when they are specifically designed to do so as in the case of asymmetries in information (see below). For example, the typical means of testing the predictions of the partial compensation hypothesis is to “handicap” or temporarily remove one member of the pair and investigate the response by the other in broods of natural size (Harrison et al. 2009). Such tests successfully reduce (or remove) the contribution by a target parent against which the responses of the other can be measured. However, it remains unclear whether evidence for (or against) partial compensation is confounded by changing perceptions regarding the manipulated partner’s quality or state and so brood value (Harrison et al. 2009), or changing perceptions about brood demand owing to reductions in contributions by a more informed, but manipulated carer (Meade et al. 2011). Indeed, more recent studies that have used targeted playbacks of offspring begging to stimulate increases in care by one member of the pair have found that unmanipulated partners responded positively, as expected under matching but not compensation (Hinde 2005; Meade et al. 2011). Additional manipulative studies that also incorporate tests of model assumptions and competing predictions will help clarify support for or against competing models of conflict resolution in bi-parental care systems.

Here, we combine observations on natural and manipulated brood sizes along a ~1,000 m elevational gradient in the French Pyrenees to investigate support for the underlying assumptions and predictions of compensation versus matching models of bi-parental care using blue tits (*Cyanistes caeruleus*). Blue tits are small (12 g), short-lived socially monogamous passerines with modest extra-pair paternity rates (~5% to 20% across populations: (Kempenaers 1994; Charmantier and Perret 2004; García-Navas et al. 2014)). Females alone build the nest and incubate the eggs (Perrins 1979), and presumably suffer greater potential for pre-hatching costs as a consequence (Visser and Lessells 2001). Males provide little sustenance to incubating females (Bambini et al. 2019), although they do perform more territory defense (Sheldon et al. 1999; Sanz et al. 2000). Both members of the pair provision nestlings with invertebrate prey from hatching, although females also contribute significantly to brooding young nestlings (Perrins 1979) during which time they might glean more information on brood demand (Meade et al. 2011). Previous studies, based on observations following partner death (Sasvári 1986), handicapping experiments (Griffioen et al. 2019), and short-term removal experiments (Iserbyt et al. 2019) all provide supporting evidence for partial compensation response rules in blue tits.

Brood size manipulations impact both potential brood value and demand since more nestlings need more food and so beg more (Leonard et al. 2000; Neuenschwander et al. 2003; Hinde and Kilner 2006). Nevertheless, because brood size manipulations have only recently been used to investigate patterns of care (Mariette and Griffith 2015; Baldan et al. 2019; Griffioen, Müller, et al. 2019), the advantages and disadvantages of this method for the present goals need highlighting. Importantly, such manipulations can be bi-directional and temporary, allowing investigation of responses during both increases and decreases in brood demand in comparison to control days. Additionally, they enable simultaneous tests of model assumptions and predictions. As an example, the inherent assumption in partial compensation models that both pair members have comparable cost/benefit functions of care will be supported if males and females respond similarly to brood size manipulations, while the primary prediction will be supported if modest responses by one pair member are met with greater responses by the other (and *vice versa*). Further, brood size manipulations avoid potential issues with parental removal or modification (see above), and can be performed in habitats where targeted playbacks are less feasible owing to uncertainty over whether or not the non-targeted partner can hear the playback. On the other hand, an obvious drawback is that because the manipulation does not target one parent over another, care is required in generating clear predictions (see below) and in accounting for the impacts of shared environmental effects on responses, including the shared brood size (see Methods).

**Fig. 1.**
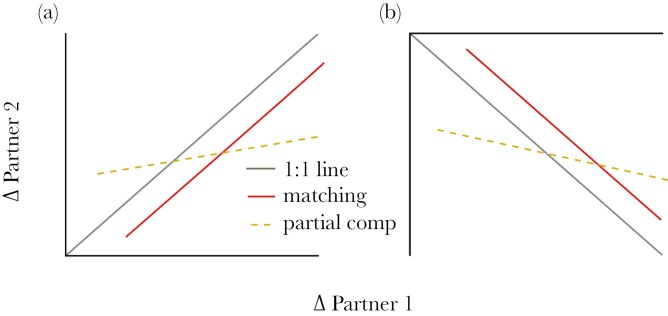
Expected relationship between changes in partner contributions on experimental days under partial compensation (yellow, dashed line) versus partner matching (red, solid line). Expected relationships are compared with the 1:1 regression line with intrecept zero (solid grey line). Analyzing changes in contributions between experimental and control observation days helps to reduce shared-environment effects, but we still expect generally positive changes (Δ) when brood sizes are enlarged (a) and negative changes (Δ) when brood sizes are reduced (b). Under partial compensation, we expect the regression slope of partner changes to differ from and cross the 1:1 line. Under matching, we assume that partner 1 on the x-axis has *more* information on brood value/demand than their less informed partner 2 on the y-axis. Asymmetries in information on brood demand have been shown to arise where one sex or individual provisions the brood more (Hinde 2005) or spends more time brooding (Meade et al. 2011). A key prediction of partner matching model is that less informed partners will follow their more informed partners in their contributions (Johnstone and Hinde 2006). Consequently, under matching, we expect changes by the less informed partner 2 to approximate (but not exceed) changes by the more informed partner 1, and so follow (but not exceed) a 1:1 regression slope.

Here, we first test the model assumptions of matching by investigating whether differences exist between pair members in the time spent with nestlings (i.e. duration of nest visits) or in the number of interactions with nestlings (i.e. provisioning rates) on control days, and so the potential for asymmetries in information on brood demand. Second, we test the assumptions of compensation models by investigating whether the two sexes show comparable responsiveness to our bi-directional brood size manipulations. Finally, we test the competing predictions of matching and compensation models by analyzing the relationship between changes in provisioning rates by each member of the pair in response to brood size manipulations after controlling for brood size and provisioning rates on control days. In this case, theory (Johnstone and Hinde 2006) and empirical work (Hinde 2005) demonstrate that the more informed individual should contribute more overall and be more responsive to manipulation, than the less informed individual. We therefore predicted that support for matching requires that the less informed sex or individual matches the response by the more informed member of the pair, leading to a regression slope for changes in provisioning rates by each member of the pair approximating 1:1 (see also Methods). By contrast, under partial compensation, the same regression slope is expected to be significantly positive in our experiment, since both members of the pair are expected to respond similarly in their shared environment, but we expect the gradient of the slope to differ significantly from (and cross) the 1:1 line because modest responders should have higher responding partners (and *vice versa*) (see Fig. 1).

## Methods

### Population and observations

This study was conducted over 2 consecutive breeding seasons (2015 to 2016), near the Station for Theoretical and Experimental Ecology in Moulis (SETE, UAR 2029, 42°57ʹ29ʹʹ N, 1°05ʹ12ʹʹ E) in the French Pyrenees. Our field sites comprise 14 mixed deciduous woodlots separated by small fields for livestock situated along an elevational gradient ranging from 430 to 1,530 m. The elevational range is associated with up to a 5 °C decrease in mean daily (24 h) temperature during the breeding season (Bründl et al. 2020). Mixed deciduous woodland dominates in all sites, although oak (*Quercus* spp.) increasingly gives way to beech (*Fagus sylvatica*) as elevation increases (Lejeune et al. 2019). The breeding density of blue tits declines with increasing elevation, and although breeding phenology is also increasingly delayed, there are no obvious changes in clutch size (mean = 8, range = 4 to 12) or fledging numbers across the elevational gradient (Bründl et al. 2020). Nevertheless, because temperature is lower and more variable, we might expect the costs of parental care, and so sexual conflict, to be exacerbated at higher elevations.

In our population, blue tits breed in Woodcrete Schwegler 2M boxes (32 mm diameter entrance holes; Schorndorf, Germany), which are placed at ~50 m intervals within each woodlot (*N* = ~650 nest boxes). For the purposes of this study, we obtained data on brood size and age with precision due to nest checks every 1 to 5 d (daily at key times, such as laying and hatching). Adults not previously ringed (winter mist-net captures or previous breeding seasons) were trapped on the nest using standard nest-box traps when chicks were at least 8 d old, usually 10 to 11; before the onset of provisioning observations and the brood size manipulation. Adults were given a unique combination of color rings and fitted with a numerically unique metal ring from the French bird ringing office (CRBPO). Females were identified by the presence of a brood patch.

Provisioning data were collected by filming nests at a distance of ~10 m for 3 h at a time using a camouflaged video camera (Sony HDR-CX220E Handycam Camcorders; Shanghai, China) (see below for sample sizes). Recordings were made when broods were 14 d old on average (± 1.2 SD; range: 11 to 17 d), with fledging occurring modally on day 21 (range 19 to 24 d) post-hatching. The age range of broods during observations thus encompassed periods of reduced brooding by mothers and maximal provisioning rates by both partners (but see below and Results). Natural brood sizes averaged 6 (± 1.5 SD), with a range from 3 to 10 nestlings. The elevation of nests used in this study averaged 670 m (SD = 192 m, range = 430 to 1,130 m). Video recordings enabled hourly visitation rates and broad prey types to be determined for each individual at each nest. The single prey item delivered in each visit was either classified as a large item (Lepidopteran caterpillars) or less well-definable, small arthropods (e.g. spiders; García-Navas et al. 2012).

### Experimental design

Nests of similar age (0 to 2 d difference) and elevation were paired for reciprocal brood swaps. The brood size manipulation was conducted over 4 consecutive days, with the first and last days serving as natural brood size controls. Provisioning rates on each control day were averaged to ensure that we removed fine-scale effects of treatment order and/or chick age effects (females control 1 mean: 20 feeds per hour ± 8 SD; control 2 mean: 23 feeds per hour ± 7 SD Welch’s *t*-test (*N* = 50,47): *t*_*94.96*_ = −1.73, *P *= 0.087; males: control 1 mean: 23 feeds per hour ± 9 SD; control 2 mean: 22 feeds per hour ± 9 SD; Welch’s *t*-test for sexes combined: *t*_*95*_ = 0.65, *P *= 0.52; *N* = 42 nests), save for the 13 instances when equipment failure or attempt failure precluded our ability to obtain observations on both control days. Performing reciprocal brood swaps over 2 d ensures that broods receive both reduction and enlargement treatments and removes any systematic bias of order effects. The pre-manipulation control video was obtained on day ~12 post-hatching (± 0.6 SD; range: 11 to 14 d), after which the nestlings were color-ringed and 2 nestlings from one of the nests (determined randomly) were fostered to a neighboring nest. The next day (day 13), the resulting increased and decreased broods were video-recorded simultaneously for 3 h, before 4 nestlings from the increased brood were fostered to the previously reduced brood (i.e. 2 chicks moved back to their original nest + 2 from the foster nest). Then on the next day (day 14), the pairs of nests were video-recorded for 3 h, and the 2 foster nestlings were removed back to their original nest. Finally, on the 4th day (day 15), a final control video of 3 h was obtained for each nest. Cross-fostering rarely took more than 30 min, and during this time, nestlings were protected in cotton bags and placed close to the body to retain warmth. Videos started on average at ~11 am on each day (± 1:56 SD), and so parents had ~15 h of daylight to become accustomed to the new brood size/demand following manipulation before recording on the subsequent day. Overall, we obtained data on provisioning rates for 47 to 51 nests of each treatment across the 2 years, with slight variation owing to video or nest failure during the experiment. We analyzed the latter 2 h of each 3 h video in order to reduce any impacts of disturbance caused by setting up the video camera.

Work was conducted under animal care permits from the French bird ringing office (CRBPO; program 576), the state of Ariège animal experimentation review (Préfecture de l’Ariège, Protection des Populations, n°A09-4), and the Région Midi-Pyrenées (DIREN, n°2013-02).

### Statistical analyses

Statistics were performed in *R* 3.4.2 (R Core Team 2023). The distributions of dependent variables were inspected visually for normality and transformed if necessary (see below) (Zuur et al. 2009). Linear models were performed in the *stats* package, and mixed models (LMMs) were performed with the *lme4* package to account for repeated measures of individuals and/or pairs (Bolker et al. 2009; Bates et al. 2015). Models were checked for overdispersion and heteroscedasticity in residuals. Quadratic effects were tested using the “poly(x,2)” function to account for the correlation between linear and quadratic functions (Chambers and Hastie 1992). Models contained terms of interest as well as potential confounders (see below for more information on specific models), although confounders were removed using a backward elimination procedure if they failed to have significant explanatory power based on changes in deviance using the ANOVA function in *R* (significance set at α < 0.05). Similarly, although we fitted a number of specific interactions (see below), based on the same criteria, these were also removed if not of primary interest and non-significant (Zuur et al. 2009).

### Sex differences on control days: testing assumptions of matching

Information on brood demand can be gleaned during nest visits, with the sex visiting the nest for longer and/or more frequently potentially gaining more information. To investigate sex differences in the frequency and/or duration of nest visitations on control days, we formed 2 linear mixed effects models. In the first, the mean duration of each nest visit across the 2 control days was fitted as the response term, whereas in the second, we fitted the average provisioning rate of each individual across the 2 control days as the response term. We inverse-transformed the mean duration of each nest visit to generate normally distributed residuals. In each model, nest box identity within each year was fitted as a random term to account for the fact that partner nest visitations are not expected to be independent. Explanatory terms included the sex of the parent, natural brood size (linear and quadratic terms to account for nonlinear effects), brood age, lay date, elevation, and year. About 90% of the single prey items delivered by parents on each nest visit in our population comprise small invertebrates (principally spiders (García-Navas et al. 2012)), with the remainder being considerably larger Lepidoptera caterpillars. The proportion of caterpillars delivered was consequently also fitted as a covariate in the control provisioning rate analysis to account for any qualitative variation in the size of prey items delivered among nests (Wright et al. 1998; Hinde 2005). Finally, in the control provisioning analysis, we also tested for interactions between sex and natural brood size, and sex and elevation, to elucidate whether sex differences in contributions were influenced by opportunities for current fitness returns and ecology.

### Testing assumptions of partial compensation: sex differences in responses

Models of partial compensation assume that the two sexes have comparable cost-benefit functions of contributing to the measured form of care. To investigate the response of each sex to the 2 treatments, we subtracted the average provisioning rate of each sex across the 2 control days from their provisioning rates on each experimental day. This generated generally positive change values on brood size increase days and negative change values on reduction days. The resulting changes were fitted as the response term in a mixed model, with both nest identity and individual identity fitted as random intercepts to account for reduced independence of individual responses by members of the same pair as well as repeated measures of individual across treatments. Here, the primary explanatory term of interest was the sex × treatment interaction, while the provisioning rate of each individual across the 2 control days, natural brood size (linear and quadratic), brood age, prey type (see above), lay date, elevation, and year were fitted as covariates.

### Response rules to partner contributions

Using brood size manipulations to investigate partner response rules is more challenging than conventional methods targeting a single pair member because both members of the pair are expected to change their provisioning rates in the same direction in response to the manipulations owing to strong shared environment effects. Reducing the impact of the shared brood size environment on responses requires analyzing the changes in provisioning rates between control and experimental days for each member of the pair whilst controlling for parameters that might be expected to affect such changes to minimize regression-to-the-mean effects (Kelly and Price 2005). To test these predictions of matching and compensation models, we performed 2 sets of 2 linear models in R.

In the first pair of analyses, we fitted the change in provisioning rates by males as the response term and their female pair member as the primary explanatory term separately on brood enlargement and reduction days. In this pair of analyses, we assumed females to be more informed about brood demand than males because they spent more time brooding (see Results; (Meade et al. 2011)). In each analysis, potential confounding predictors of change were fitted as co-variates, including natural brood size (linear and quadratic), average provisioning rates across control days (of females), and the difference in average provisioning rates on control days between males and females. In the second pair of analyses, we replaced sex with the top versus bottom provisioners on control days, under the assumption that information on brood demand is primarily gleaned when broods are begging for food on nest arrival (Kilner and Johnstone 1997). In these analyses, the change in provisioning rate of the bottom provisioner on control days was fitted as the response term, and the change by the top provisioner on control days was fitted as the primary explanatory term, again in separate analyses for brood enlargement and reduction days. The same covariates were fitted as outlined above for the sex-specific analyses, but where average control rates of top provisioners, and the difference between top and bottom (control day) provisioners, replace sex. Control brood size was fitted to control for the shared environment of brood demand, since broods with more offspring need more food, while provisioning rates on control days were fitted to test (and control for) potential regression-to-the-mean effects (Kelly and Price 2005; see Discussion).

Interpreting support for compensation versus matching requires a clear understanding of the expected results under each in this novel experimental approach. Most importantly, although we fit control-day brood size and control-day provisioning rates as covariates, we are not able to remove all shared environment effects. For example, metrics of parenting “ability” might vary more among versus within pairs, and both members of the pair obviously experience similar begging intensity by their brood and share the same territory. Thus, under both matching and partial compensation, responses to brood size manipulation are expected to covary positively within the pair. Nevertheless, in their model, Johnstone and Hinde (2006) showed that more informed individuals should both be more responsive to changes in brood demand and contribute more overall (when demand increases), than less informed partners. In addition, Hinde (2005) showed empirically in great tits that more informed individuals contributed more to nestling provisioning than less informed partners. Thus, under matching, we would expect any changes by the less informed member of the pair to follow, but not exceed changes by the more informed partner on average. In effect, therefore, perfect matching would predict a 1:1 relationship (with intercept zero) between changes by the less informed pair member against changes by the more informed pair member (Fig. 1). By contrast, under partial compensation, the same regression line should cross the 1:1 line, since small changes by one sex or individual should be met with relatively greater changes by the other, and vice versa for high responders (Fig. 1). Hence, in a final set of analyses, we tested whether the slope estimates of partner responses differed significantly from 1:1, as expected under partial compensation but not matching (Zar 1999).

## Results

### Sex differences on control days: testing assumptions of matching

**Fig. 2.**
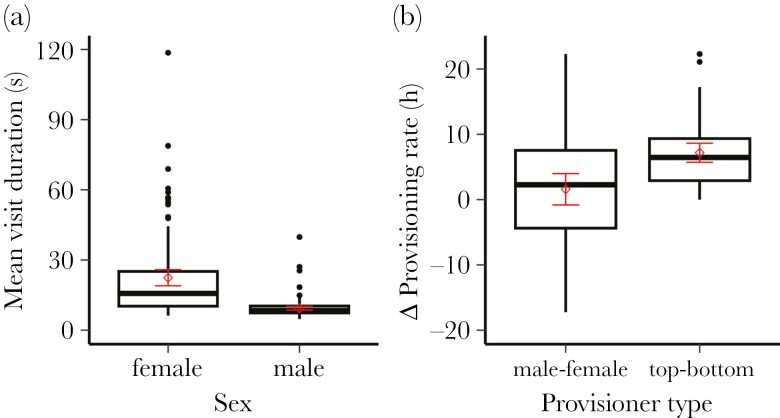
Differences between sexes in relation to (a) nest visit duration (s) and (b) provisioning rate (h), and top versus bottom provisioners in provisioning rates (h). Represented are differences in provisioning rates between experimental and control days (Δ). Boxplots are based on raw data, including the interquartile range, median, minimum and maximum range and outliers. The mean is represented (empty circle) with accompanying standard error bars in red.

Although brooding has ceased by day 11 in blue tits, information can still be gained by spending protracted time on the nest during provisioning visits. The average duration of nest visits during feeding on control days varied from a low of 5 s to a high of 114 s (median = 10, IQR = 8 to 19 s). This duration declined with increasing brood age from day 11 when observations began and in large brood sizes, but there were no effects of lay date, elevation, or year (Table 1). Further, we found that females spent twice as long in the nest box during feeding visits than their male partners (χ^2^ = 124.303, *P* < 0.001; Fig. 2A). Indeed, in 49 of 54 pairs (> 90%), females had longer visit durations than their male partners. Previous studies in blue tits show females perform more nest sanitation than males in old broods, explaining the greater nest visit duration and potential to gain information (Bańbura et al. 2001). These results suggest that if information on brood demand is gleaned by spending more time on the nest, females are the more informed sex.

**Table 1.** Results of a LMM examining mean visit duration (s) on control days, after reciprocal (1/y) transformation. *P* values are derived from likelihood ratio tests based on chi-square (χ^2^) values (*N* = 108).

Predictors	Reference category	Estimate	± SE	*t* value	DF	χ^2^	*P* value
Intercept		−0.057	0.057	−0.999	-	-	-
Sex (Male)	Female	0.055	0.005	11.149	1	124.303	<0.001
Natural brood size (linear)		0.130	0.030	4.276	-	-	-
Natural brood size (quadratic)		−0.061	0.031	−2.007	2	22.019	<0.001
Brood age		0.010	0.005	2.138	1	4.573	0.032
Julian lay date		0.000	0.001	0.480	1	0.231	0.631
Elevation		0.000	0.000	−0.918	1	0.842	0.359
Year (2016)	2015	−0.005	0.006	−0.844	1	0.713	0.399

Alternatively, information might be gleaned primarily at nest arrival when nestlings are begging most intensively (Kilner and Johnstone 1997), in which case the individual with the higher provisioning rate on control days should have more information. Individual provisioning rates on control days averaged 22 feeds h^−1^ (SD = 7.4, range = 7.6 to 46.6). Natural variation in provisioning rates increased as a linear function of increasing brood size and declined as a function of an increasing proportion of feeds comprising caterpillars and elevation but was unaffected by lay date or year (Table 2). After controlling for these effects, we found no evidence for sex differences in provisioning rates, either as a main effect (Fig. 2B, left box plot), or in interaction with brood size or elevation (Table 2). Nevertheless, it is conceivable that individuals within pairs differ significantly in their provisioning rates, even if not linked to their sex. Indeed, the absolute difference in control provisioning rates between pair members averaged 7.2 feeds h^−1^ (SD = 5.4; Fig. 2B, right box plot), suggesting that if information is gained at nest entry, pair members might differ significantly in their information on brood demand independently of their sex.

**Table 2.** Results of an LMM examining mean provisioning rate (h). *P* values are derived from likelihood ratio tests based on chi-square (χ^2^) values (*N* = 110).

Predictors	Reference category	Estimate	± SE	*t* value	DF	χ^2^	*P* value
Intercept		18.472	4.202	4.397	-	-	-
Sex (Male)	Female	1.585	1.200	1.320	1	1.744	0.187
Natural brood size (linear)		1.754	0.468	3.751	1	14.074	<0.001
Natural brood size (quadratic)		−5.999	7.021	−0.854	1	0.797	0.372
Sex (Male) × Natural brood size (linear)	Female × Natural brood size (linear)	−0.537	0.847	−0.633	1	0.401	0.527
Sex (Male) × Natural brood size (quadratic)	Female × Natural brood size (quadratic)	−2.045	12.863	−0.159	1	0.678	0.713
Sex (Male) × Elevation		−0.007	0.006	−1.182	1	1.398	0.237
Proportion caterpillar		−10.907	4.851	−2.248	1	5.056	0.025
Brood age		0.123	0.749	0.164	1	0.027	0.870
Julian lay date		0.006	0.177	0.034	1	0.001	0.973
Elevation		−0.009	0.004	−2.423	1	5.871	0.015
Year (2016)	2015	0.438	1.389	0.315	1	0.099	0.753

### Testing assumptions of partial compensation: sex differences in responses

A key assumption of partial compensation models is that the 2 members of each pair have comparable cost-benefit functions of investment in the care behavior observed. To test this assumption, we analyzed sex differences in changes to provisioning rates between the 2 treatment days relative to the average rates across the 2 control days (Table 3). On average, pairs increased their feeding rates by 18% on brood enlargement days (mean = 26 feeds/h ± 8.8 SD) and reduced it by 23% on reduction days (mean = 17 feeds/h ± 8.4 SD). The magnitude of these changes was significantly impacted by negative effects of control provisioning rate and the proportion of caterpillars delivered, and there was also a trend for brood age to negatively affect changes (Table 3). After accounting for these effects, we found a significant independent effect of treatment (*P *< 0.001) and a trend for sex differences (*P *= 0.054), with males tending to provision slightly more frequently than females (Table 3). However, importantly for the assumptions of compensation models, we failed to find evidence of a significant interaction between treatment and sex on changes in provisioning responses (*P *= 0.32)—suggesting that both members of the pair have comparable response rules to variation in brood demand or value

**Table 3.** Results of an LMM examining the effect of the brood size manipulation on the difference in provisioning rates (h) between experimental and control days. *P* values are derived from likelihood ratio tests based on chi-square (χ^2^) values (*N* = 200).

Predictors	Reference category	Estimate	± SE	*t* value	DF	χ^2^	*P* value
Intercept		1.434	1.704	0.841	-	-	-
Sex (Male)	Female	1.799	0.934	1.926	1	3.708	0.054
Treatment changes (Increases)	Decreases	9.125	0.931	9.801	1	96.059	<0.001
Natural brood size (linear)		0.170	0.360	0.473	1	0.224	0.636
Natural brood size (quadratic)		2.856	7.242	0.394	1	0.186	0.667
Sex (Male) × Treatment changes (Increases)	Female × Decreases	−1.798	1.827	−0.984	1	0.968	0.325
Control rate		−0.246	0.066	−3.737	1	13.968	<0.001
Proportion caterpillar		−9.662	2.970	−3.253	1	10.585	0.001
Brood age		1.239	0.637	1.944	1	3.779	0.052
Julian lay date		0.011	0.133	0.080	1	0.007	0.936
Elevation		0.002	0.003	0.547	1	0.299	0.584
Year (2016)	2015	−1.343	0.980	−1.370	1	1.876	0.171

### Testing the predictions of matching versus compensation

**Fig. 3.**
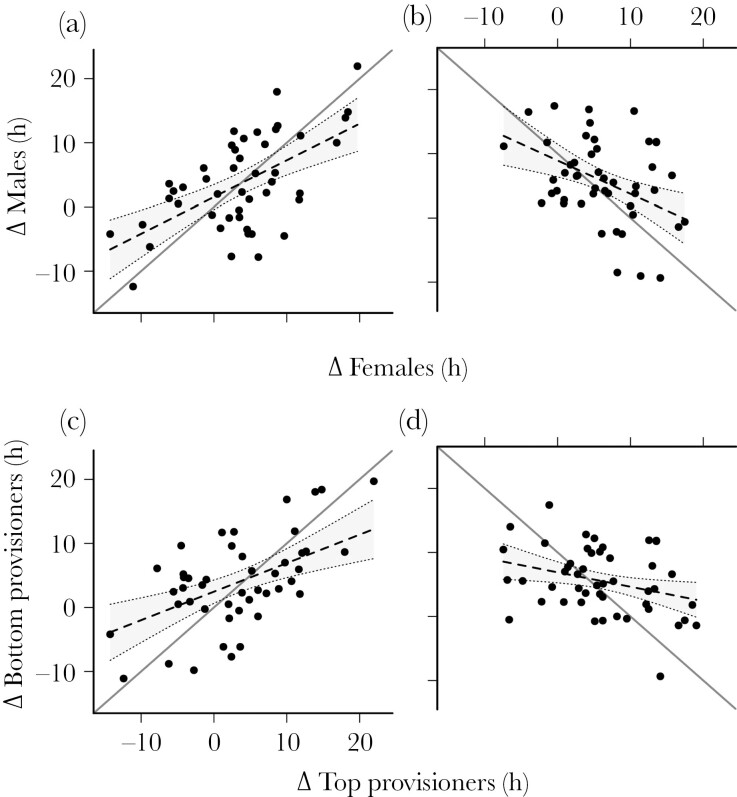
Differences in experimental versus control provisioning rate (h). Shown are these provisioning differences (Δ) between females and males in (a) enlarged and (b) reduced treatments, and top versus bottom provisioners in (c) enlarged and (d) reduced treatments. Represented are predicted lines of best fit (dashed lines with accompanying 95% confidence intervals) and raw data points.

Given that females spend more time with the brood than males (Table 1), they should have more information regarding brood demand (Meade et al. 2011). Despite this, we found little evidence that males match the changes in provisioning contributions by female partners during brood size manipulations. For example, on brood enlargement days, 49% of males increased their contributions more than their female partner, and 66% contributed more overall, while on brood reduction days, 40% reduced their contributions more than their female partner, and 35% contributed less overall. Consequently, after considering potential effects of brood size, female control provisioning rates, and the difference in provisioning rates between the pair on control days (Tables 4 and 5), we found that the regression slope between changes in provisioning rates by females versus males on both enlargement (Table 4, Fig. 3A) and reduction (Table 5, Fig. 3B) days clearly crossed the 1:1 line as expected under partial compensation but not matching (slope differences from 1:1: brood enlargement days: *t*_45_ = 4.23, *P* < 0.001; brood reduction days: *t*_46_ = 3.35, *P* = 0.0023).

**Table 4.** Results of an LM examining the effect of the change in female provisioning rate (h) on the change in male provisioning rates (h) between increase and control days. P values are derived from *F*-tests based on *F* values (*N* = 47).

Predictors	Estimate	± SE	*t* value	DF	*F*	*P* value
Intercept	4.048	4.133	0.979	-	-	-
Change rate females	0.453	0.134	3.383	1	11.444	0.002
Control rate females	−0.088	0.179	−0.490	1	0.241	0.626
Sex difference control rate	−0.087	0.122	-0.714	1	0.510	0.479
Natural brood size (linear)	−12.151	7.049	−1.724	1	2.831	0.100
Natural brood size (quadratic)	3.034	6.371	0.476	1	0.227	0.637

**Table 5.** Results of an LM examining the effect of the change in female provisioning rate (h) on the change in male provisioning rates (h) between decrease and control days. *P* values are derived from *F*-tests based on *F* values (*N* = 48).

Predictors	Estimate	± SE	*t* value	DF	*F*	*P* value
Intercept	−7.511	3.825	−1.963	-	-	-
Change rate females	0.447	0.165	2.710	1	7.344	0.010
Control rate females	0.391	0.175	2.233	1	4.985	0.031
Sex difference control rate	0.363	0.104	3.475	1	12.079	0.001
Natural brood size (linear)	−10.240	7.296	−1.403	1	1.525	0.224
Natural brood size (quadratic)	9.938	5.728	1.735	1	3.010	0.090

An alternative is that individuals that provision at high rates on control days have more information, irrespective of their sex. However, again, there was little evidence to suggest that low provisioners on control days matched the responses of high control-day provisioners on experimental days. In this case, 49% of low provisioners on control days responded more on brood enlargement days than their higher control-day provisioning partner, while 46% of lower provisioning individuals on control days responded more than their higher contributing partner during brood reductions. Thus, the relationship between partner responses clearly crossed the 1:1 line on both enlargement and reduction days (slope differences from 1:1: brood enlargement days: *t*_45_ = 4.83, *P* < 0.001; reductions: *t*_46_ = 8.42, *P* < 0.001; Fig. 3C,D). These results were found despite controlling for potential effects of brood size, female control provisioning rates and the difference in provisioning rates between the pair on control days (Tables 6 and 7).

**Table 6.** Results of an LM examining the effect of the change in top provisioner rate (h) on the change in bottom provisioner rates (h) between increase and control days. *P* values are derived from *F*-tests based on *F* values (*N* = 47).

Predictors	Estimate	± SE	*t* value	DF	*F*	*P* value
Intercept	7.443	3.780	1.969	-	-	-
Change rate top provisioner	0.416	0.121	3.441	1	11.843	0.001
Control rate top provisioner	−0.231	0.170	−1.358	1	1.845	0.182
Provisioner difference control rate	0.160	0.221	0.724	1	0.524	0.473
Natural brood size (linear)	−1.027	0.664	−1.546	1	2.392	0.130
Natural brood size (quadratic)	4.787	6.022	0.795	1	0.632	0.431

**Table 7.** Results of an LM examining the effect of the change in top provisioner rate (h) on the change in bottom provisioner rates (h) between increase and control days. *P* values are derived from *F*-tests based on *F* values (*N* = 48).

Predictors	Estimate	± SE	*t* value	DF	*F*	*P* value
Intercept	6.501	2.918	2.228	-	-	-
Change rate top provisioner	0.225	0.092	2.446	1	5.983	0.019
Control rate top provisioner	0.372	0.126	2.940	1	8.641	0.005
Provisioner difference control rate	−0.609	0.151	−4.045	1	16.363	0.000
Natural brood size (linear)	−1.378	0.488	−2.824	1	7.974	0.007
Natural brood size (quadratic)	6.214	4.485	1.386	1	1.920	0.173

## Discussion

We found significant support for the assumptions of both matching and partial compensation models. In support of the key assumption of matching (Johnstone and Hinde 2006), females spent twice as much time in contact with the nestlings, and so had the potential to gain more information about brood demand (Meade et al. 2011). In addition, males and females were similarly responsive to brood size alterations, in support of the assumption of partial compensation models that parents have similar cost-benefit provisioning curves (Houston and Davies 1985; McNamara et al. 1999; Johnstone and Hinde 2006). Despite support for the key assumption of both models, we only found support for our predictions of partial compensation. Notably, on experimental days, we found little evidence to suggest that males matched the changes by their potentially more informed female partners. Likewise, if feeding visits provide information, low control-day provisioners did not match the changes of their potentially more informed higher control-day provisioning partners. Instead, individuals showing modest changes tended to have partners showing more extreme changes, irrespective of their sex. This pattern generated a relationship between responses by each member of the pair that clearly crossed the 1:1 regression line, in support of compensation rather than matching. Our results suggest that partial compensation response rules predominate during nestling provisioning in this population of blue tits, despite the potential for asymmetry in information about brood demand between the parents.

Previous tests of compensation and matching models have typically targeted one member of the pair and investigated responses by the other. For example, by reducing the contributions of one member of the pair through tail weighting, wing-feather clipping, or hormonal implantations, several studies have provided general evidence for partial compensation response rules by the unmanipulated partner (Harrison et al. 2009). On the other hand, in their meta-analysis, Harrison et al. (2009) also revealed exceptions, with the type of experiment performed and the sex on which it was performed influencing the magnitude of responses by those unmanipulated. Such inconsistencies might be due to changes in the perception of partner quality following experimental manipulation (Harrison et al. 2009) or asymmetries in information about brood demand or value (Johnstone and Hinde 2006). Indeed, when such asymmetries were taken into consideration in targeted playback experiments, wherein one member of the pair experienced artificial increases in begging intensity, matched, rather than compensatory, response rules have been observed (Hinde 2005; Meade et al. 2011). Taken together, these studies suggest that tests of model assumptions need to be integrated into tests of model predictions in future experiments and that direct tests of the competing predictions of matching and partial compensation within the same experimental framework would help to clarify the relative support for each model.

We performed temporary, bi-directional brood size manipulations, which allowed us to test key assumptions and predictions of both compensation and matching models simultaneously, whilst accounting for natural parental care levels on control days. However, because a specific parent was not targeted, care is required when formulating specific predictions and devising appropriate analyses with due consideration of confounding shared-environment effects. For example, a positive correlation is expected between the pair in response to a common brood size manipulation because both members of the pair experience the same brood size, begging intensity, and territory quality (Hinde and Kilner 2006). Given these considerations, and the inability to fully control for all of them, we predicted that responses by pair members would positively covary (i.e. both increasing in response to brood size enlargement and both decreasing in response to brood size reductions). However, we reasoned that support for matching would be manifest as a regression slope of changes in provisioning rates between the more versus less informed partners approximating 1:1, since changes by less informed partners should seldom exceed the changes by more informed partners (Hinde 2005; Johnstone and Hinde 2006). By contrast, under partial compensation, although we would still expect a positive association between changes in the provisioning rate of pair members (see above), the same regression line should cross 1:1 because modest responders should have more extreme responding partners (and *vice versa*). Irrespective of whether or not we compared the responses as a function of sex or control provisioning rates, regression lines between partner responses crossed and deviated significantly from 1:1, thereby suggesting predominant support for partial compensation, rather than matching.

On the other hand, evidence for partial compensation might also be expected by chance through regression-to-the-mean effects—high responders should have lower responding partners and vice versa, by chance alone (Kelly and Price 2005). The degrees to which regression to the mean effects confound studies of partial compensation are unclear. In long-tailed tits (*Aegithalos caudatus*), low provisioners on control days increased their provisioning rates more in response to experimental increases in nestling begging rates than did high provisioners on control days (Meade et al. 2011). In our study, we attempted to minimize the confounding influences of regression-to-the-mean effects, by controlling for natural brood size, the provisioning rate of the individual on the x-axis on control days, as well as the difference in provisioning rates between the pair members on control days. Further, we reran our analyses after removing the top and bottom 10% of responders on the x-axis to reduce the impact of extreme values that will be most influenced by regression-to-the-mean phenomena. However, again our previous results were maintained: modest responders had more extreme responding partners. Finally, if regression-to-the-mean effects were confounding our results, we would expect comparable average slopes to those we observed (see Fig. 3A–D) when a change by parent 2 is regressed with 100 random partner 1’s from the population (Baldan et al. 2019). On the contrary, in all analyses (i.e. for both increase and decrease treatments when partner 1 was female on control days), the regression slopes were flat (mean *r* values = 0.001 (95% CI [−0.030, −0.059]) for increases and 0.041 (95% CI [0.016, 0.071]) for decreases) (simulation data as excel worksheet in Supplementary Material). Thus, whilst regression-to-the-mean effects are difficult to discount entirely, we suggest that such effects are unlikely to confound our primary conclusions.

In conclusion, we suggest that our results are more consistent with partial compensation, rather than matched, response rules in this population of blue tits. In doing so, we do not rule out entirely a role for partner-based information on provisioning rates. For example, that pairs show a positive correlation in response rules more so than expected by chance might hint at some role of partner matching (Baldan et al. 2019), although an alternative interpretation is that each partner is responding to changes in brood demand rather than each other. Additionally, in previous experiments where females were induced to lay 1 to 2 more eggs but where pairs were not forced to provision more offspring (additional eggs were removed), resulting increases in female provisioning rates were not associated with reductions in male care, as might be expected if only compensation mechanisms were operating (Bründl et al. 2019). Nevertheless, our conclusions, using a novel experimental approach, corroborate findings based on partner death (Sasvári 1986), handicapping experiments (Griffioen, Iserbyt, et al. 2019), and short-term removal experiments (Iserbyt et al. 2019) in other populations of blue tits. Why firm evidence for matched responses was not detected, given the apparent scope for asymmetries in information between pair members, is not clear. One possibility is that asymmetries were not sufficient to drive an effect (Hinde and Kilner 2006; Johnstone and Hinde 2006). For example, Meade et al. (2011) found that in long-tailed tits matching effects were particularly strong early in the nestling phase when females spend most of their time brooding. However, Johnstone and Hinde (2006) suggested that weaker asymmetries in information about brood demand should lead to stronger compensation responses by the more informed sex. Given that we do not find evidence for this suggests that any potential differences in information were not used to influence partner response rules. Another possibility is that asymmetries in information on control days break down on experimental days. This possibility seems unlikely to us, especially on brood reduction days when females spend even more time in contact with nestlings, and so information asymmetries should be greater. Alternatively, perhaps directly monitoring the contributions of a more informed partner is challenging in most populations of blue tits, with parents using changes in begging intensity to underpin provisioning decisions rather than partner behavior. Admittedly difficult to achieve, future experiments are needed to determine how information gained is used and to quantify the magnitude of actual information asymmetries between pair members. Further, one could investigate potential mediation mechanisms of parental care response rules such as coordination and conditional cooperation using similar experimental setups as presented in this study. Overall, our findings highlight the potential complexity of compensatory responses in stabilizing parental care at the nest and advance our understanding of the evolution of bi-parental care behavior.

## Supplementary Material

arae059_suppl_Supplementary_Data

## Data Availability

Analyses reported in this article can be reproduced using the data provided by Bründl et al. (2024).
